# 

**Published:** 1965-10

**Authors:** 


INCORPORATING THE MEDICAL JOURNAL OF THE SOUTH WEST
Vol 80 (iv) No 298

